# Increased expression of *Drosophila Sir 2* extends life span in a dose-dependent manner

**DOI:** 10.18632/aging.100599

**Published:** 2013-09-07

**Authors:** Rachel Whitaker, Shakeela Faulkner, Reika Miyokawa, Lucas Burhenn, Mark Henriksen, Jason G. Wood, Stephen L. Helfand

**Affiliations:** Department of Molecular Biology, Cell Biology and Biochemistry, Division of Biology and Medicine, Brown University, Providence, RI 02912, USA

**Keywords:** aging, life span, Sir2, sirtuin, SIRT1, dnaJ-H, dose response, Drosophila melanogaster, deacetylase, HDAC

## Abstract

Sir2, a member of the sirtuin family of protein acylases, deacetylates lysine residues within many proteins and is associated with lifespan extension in a variety of model organisms. Recent studies have questioned the positive effects of Sir2 on lifespan in *Drosophila*. Several studies have shown that increased expression of the Drosophila Sir2 homolog (*dSir2*) extends life span while other studies have reported no effect on life span or suggested that increased *dSir2* expression was cytotoxic. To attempt to reconcile the differences in these observed effects of *dSir2* on Drosophila life span, we hypothesized that a critical level of *dSir2* may be necessary to mediate life span extension. Using approaches that allow us to titrate *dSir2* expression, we describe here a strong dose-dependent effect of *dSir2* on life span. Using the two transgenic *dSir2* lines that were reported not to extend life span, we are able to show significant life span extension when *dSir2* expression is induced between 2 and 5-fold. However, higher levels decrease life span and can induce cellular toxicity, manifested by increased expression of the JNK-signaling molecule Puc phosphatase and induction of *dnaJ-H*. Our results help to resolve the apparently conflicting reports by demonstrating that the effects of increased *dSir2* expression on life span in *Drosophila* are dependent upon *dSir2* dosage.

## INTRODUCTION

Silent Information Regulator 2 (Sir2) is an NAD^+^ -dependent deacetylase that is highly conserved from bacteria to mammalian systems. While yeast Sir2 was originally reported to deacetylate histones, the mammalian Sir2 homologs (SIRT1-7) have since been shown to deacetylate a wide variety of other proteins including p53, Foxo, PGC-1α, Ku70 and NF- κB and play key roles in age-related diseases [1]. Increased expression of sirtuins has been found to increase life span in yeast [2], *C. elegans* [3,4], *Drosophila melanogaster* [5,6,7] and male mice [8]. Many of the identified targets of sirtuins have also been shown to have significant effects on longevity [1].

Increasing the expression of *dSir2* increases fly life span when expression is induced from the endogenous locus by either the ubiquitous Tubulin-Gal4 driver or the neuron-specific ELAV-Gal4 driver [5]. Importantly, life span is also increased when increased expression of *dSir2* is temporally restricted to adulthood and spatially to either neurons [5,6] or abdominal fat body [7] by use of the Gene Switch system. The Gene Switch system has the advantage of allowing selective expression in adults via the presence of the inducing agent, RU-486, in the fly food. Therefore, genetically identical siblings from the same cohort as the experimental flies that are not induced with RU-486 can serve as controls, thereby eliminating any potential concern regarding genetic background effects on life span. In these studies [5,6,7], increased *dSir2* expression was mediated from the endogenous *dSir2* locus through use of a P-element mediated insertion of the UAS sequence upstream of *dSir2* to generate an “EP” line [9], allowing induced expression of *dSir2* from its endogenous locus when the GAL4 protein is present. While the most commonly used EP line is dSir2^EP2300^, other similarly constructed fly lines have also been shown to extend life span when *dSir2* expression is increased using some of the same Gal4 drivers [5].

A recent study reported no life span extension when *dSir2* was over expressed by using the constitutive, ubiquitously expressing Tubulin-Gal4 driver, either with the dSir2^EP2300^ line, or with either of two newly constructed lines containing an inducible UAS-dSir2 transgene in addition to the endogenous copy of *dSir2* [10]. The authors attributed the lack of lifespan extension to the use of more appropriate genetic background controls [5]. However, increased expression of *dSir2* in the dSir2^EP2300^ line did show extended life span when driven by the inducible neuron-specific ELAV-Gene Switch driver [5,6] and more recently by the inducible abdominal fat-body specific S_1_-106-Gene Switch driver [7], where the controls are genetically identical flies from the same cohort, ruling out genetic background as the cause for life span extension.

Another recent study found that ubiquitous or neuronal over-expression of *dSir2* from a UAS-dSir2 transgene was developmentally lethal [12]. Furthermore, expression of this UAS-dSir2 gene under control of the strong eye-specific GMR-Gal4 driver caused developmental apoptosis of cells of the eye, while simultaneously inducing JNK-signaling. The genetic locus of *dSir2* partially overlaps with that of a gene that is transcribed in the opposite direction, the *dnaJ-h*omologue (*dnaJ-H*) gene. While the specific function of DNAJ-H has not been shown, it has homology to the Heat Shock Protein 40 family of proteins, which are known to be involved in protein folding, translation, and degradation [11]. Interestingly, expression of *dSir2* from the native *dSir2* locus in the eye using the same robust GMR-Gal4 driver with the dSir2^EP2300^ line resulted in a greater than 12-fold increase in *dSir2* mRNA and a 2-fold induction of *dnaJ-H* mRNA expression [12]. From these results, the authors concluded that increased *dSir2* expression is lethal and that *dSir2* normally regulates cell survival and death in *Drosophila*. They hypothesized that the life span extension seen in the dSir2^EP2300^ line was likely a result of the simultaneous over-expression of both *dSir2* and *dnaJ-H*. In contrast, subsequent reports demonstrated that 2.5-fold, 3-fold, and 5-fold increased expression of *dSir2* from the native *dSir2* locus results in life span extension without an increase in *dnaJ-H* mRNA expression [6,7].

The conflicting results of these studies, demonstrating in one case that increased expression of *dSir2* is lethal [12], in a second that there is no effect on life span [10], and in third, fourth and fifth cases, that there is significant life span extension [5,6,7], have been a barrier to understanding the role of *dSir2* in *Drosophila* aging. In the present study, we present data showing that when dose-dependent effects of *dSir2* are taken into account, these many discrepancies can be resolved. Through a detailed series of studies in which we examine the effects of expression of *dSir2* at low, moderate and high levels on life span, we demonstrate that modest levels of *dSir2* are indeed effective at extending life span, and that high levels of expression likely induce a cellular stress response that is reflected by induction of *dnaJ-H*. Thus, our studies show that moderately increased levels of *dSir2* can extend life span in *Drosophila melanogaster*.

## RESULTS

### dSir2 expression from UAS-dSir2 transgenes can increase or decrease life span

**Figure 1:**
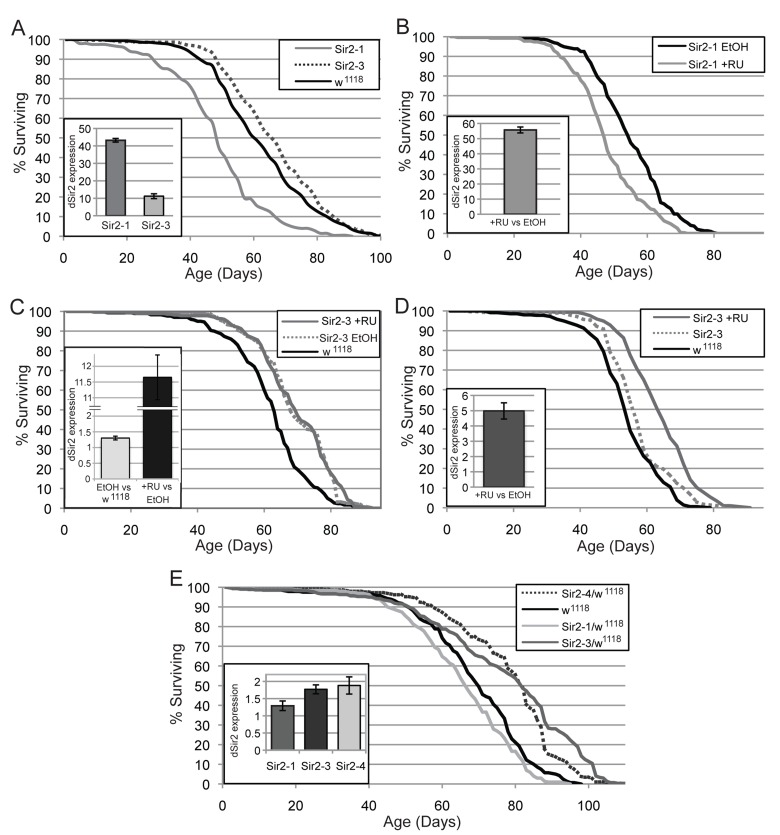
Effects of increased *dSir2* expression on life span vary under different conditions (**A**) When two different UAS-dSir2 expressing lines are driven by the Tubulin-Gal4 driver, one line extends life span (Sir2-3), while the other is detrimental (Sir2-1), compared to the control (Tubulin-Gal4>w^1118^). (**A**, inset) Sir2-1 displays very highly increased dSir2 expression, while Sir2-3 increases *dSir2* expression more moderately. (**B**) When the Sir2-1 transgene is induced to express dSir2 by the Tubulin-Gene Switch driver, life span is decreased. (**B**, inset) *dSir2* is expressed at greater than 50-fold Tubulin-Gene Switch>Sir2-1 flies. (**C**) In the Tubulin-Gene Switch>Sir2-3 flies, *dSir2* is mildly elevated and life span extended when comparing the ethanol-only condition (no RU-486) to the genetic background control. (**C**, inset) The induction of higher levels of *dSir2* from Sir2-3 using RU-486 does not further increase life span. (**D**) When the Sir2-3 transgene is expressed using the neuron-specific ELAV Gene Switch driver, life span is extended when RU-486 is used. (**D**, inset) dSir2 expression is 5-fold increased in ELAV-Gene Switch>Sir2-3 flies. (**E**, inset) Some UAS-dSir2 lines show elevated *dSir2* levels in a heterozygous state without the presence of a driver. (**E**) Fly lines that show an increase in *dSir2* levels also extend life span in the absence of a driver. Error bars represent SD of 3 biological replicates. Life span statistics can be found in table S1, and qPCR p-values in table S2.

To examine the effect of increasing *dSir2* expression on longevity, we examined the life span of flies carrying either of two newly generated UAS-dSir2 transgenes (Sir2-1 and Sir2-3) driven by the ubiquitous Tubulin-Gal4 driver. The life span of these flies was compared to Tubulin-Gal4 flies crossed to wild type flies from the same genetic background as the UAS-dSir2 transgenes. Under these conditions, Tubulin-Gal4>Sir2-3 flies showed life span extension, while Tubulin-Gal4>Sir2-1 flies had a shortened life span (Fig. 1A). The discrepancy of finding of a decrease in life span with the Sir2-1 transgene and an increase with the Sir2-3 transgene led us to measure the levels of *dSir2* expression in these flies. Due to difficulties in establishing a functional dSIR2 western protocol, we instead utilized RT-qPCR to measure mRNA levels in this study. We found that flies containing the Sir2-1 transgene over expressed *dSir2* at very high levels (~ 45-fold increase), compared to the Sir2-3 transgene (~ 11-fold increase) (Fig. 1A, inset).

To eliminate the possibility that increased expression of *dSir2* during development in the presence of the constitutively expressed Tubulin-Gal4 driver might contribute to the deleterious effect we see on adult life span in the Sir2-1 line, we utilized the RU-486 inducible Gene Switch system in which control and experimental flies are genetically identical offspring from the same cohort and increased expression can be induced in, and thus restricted only to adult life. We examined the life span of flies with increased expression of *dSir2* from the Sir2-1 transgene driven by the conditional Tubulin Gene Switch driver (Tubulin-Gene Switch). When expression of the Sir2-1 construct was induced using RU-486 in the food of adult flies, the life span of these flies was shortened, as seen for the Tubulin-Gal4>Sir2-1 life span (Fig. 1B). We measured the level of *dSir2* mRNA in these flies, and found an approximately 50-fold induction of *dSir2*, consistent with the high level of induction seen with the constitutive Tubulin-Gal4 driver (Fig. 1B, inset).

Additionally, we crossed another UAS-dSir2 line, Sir2-4, to the Tubulin Gene Switch driver and observed an increase of ~15-fold in *dSir2* levels for RU-486-induced flies compared to EtOH controls, without any observable change in median life span (Fig. S1).

Given that the level of *dSir2* induction using both the Sir2-1 and Sir2-4 transgenes in these studies was high, we hypothesized that these levels of *dSir2* might be detrimental to life span. In order to better test the effects of increased *dSir2* expression at more moderate levels, we performed life span and mRNA expression experiments using the Sir2-3 transgene, which induced *dSir2* expression more moderately than Sir2-1. We also included an additional genetic background control in this experiment, where we crossed w^1118^ flies (injection stock for the transgene lines Sir2-1, Sir2-3, Sir2-4) to the Tubulin-Gene Switch driver, and measured life span in these flies with and without RU-486 induction. Life spans of control w^1118^ flies carrying the Tubulin-Gene Switch driver was the same with or without RU-486, demonstrating that RU-486 treatment does not directly affect life span in this w^1118^ strain.

When the Sir2-3 transgene was expressed ubiquitously using the Tubulin-Gene Switch driver in the presence of RU-486, we observed no life span extension compared with genetically identical uninduced (ethanol-fed) flies. However, inclusion of the additional w^1118^ genetic background control in this study allowed for a comparison of uninduced control flies (Tubulin-Gene Switch> Sir2-3) with the w^1118^ controls. Surprisingly, we found that in comparison to the Tubulin-Gene Switch>w^1118^ controls, both the uninduced and the induced Tubulin-Gene Switch>Sir2-3 flies exhibited an extended life span (Fig. 1C). This result was unexpected, given that the absence of RU-486 in the food should have meant that there was no induction of *dSir2*. In order to clarify this result, we also examined the levels of *dSir2* mRNA in all of these flies, and found that the diluent, ethanol-fed control Tubulin-Gene Switch>Sir2-3 flies had a mild but significant elevation in *dSir2* levels (~1.5-fold induction) when compared with the Tubulin-Gene Switch>w^1118^ controls. The Tubulin-Gene Switch>Sir2-3 flies that were induced with RU-486 in the food showed an ~ 11-fold increase in *dSir2* levels (Fig. 1C, inset). This finding suggests that in these conditions, a modest ~1.5-fold and significantly higher ~11-fold elevation of *dSir2* can extend life span.

Finally, we crossed the Sir2-3 transgenic flies to the neuron-specific ELAV-Gene Switch driver and evaluated life span extension and *dSir2* mRNA levels. Again, we included the ELAV-Gene Switch>w^1118^ genetic background control to allow for comparison with the uninduced ELAV-Gene Switch>Sir2-3 ethanol controls. In this experiment, the uninduced ELAV-Gene Switch>Sir2-3 flies did not have significantly elevated *dSir2* levels compared to the ELAV-Gene Switch>w^1118^ genetic control. Consistent with this finding, we saw no life span extension for the uninduced ELAV-Gene Switch>Sir2-3. However, the RU-486 induced ELAV-Gene Switch>Sir2-3 flies had a 5-fold increase in *dSir2* mRNA levels and extended life span as compared to the uninduced Elav-Gene Switch>Sir2-3 control flies and the Elav-Gene Switch>w^1118^ control flies (Fig. 1D).

### Moderate increases in dSir2 expression from UAS-dSir2 transgenes extend life span

The ability of the Sir2-3 line to induce *dSir2* expression in the uninduced condition of the Tubulin-Gene Switch life span led us to examine whether the additional copy of *dSir2* from the UAS-dSir2 transgene may under certain circumstances express *dSir2* in the absence of Gal4. To directly test whether the presence of one copy of the UAS-dSir2 transgene could lead to increased expression of *dSir2* without the presence of a Gal4 driver, we crossed three different homozygous UAS-dSir2 transgenic lines (Sir2-1, Sir2-3, and Sir2-4) to their genetic background control (w^1118^) and measured*dSir2* mRNA levels in the F1 heterozygotes (Fig. 1E, inset). One copy of the Sir2-1 insertion (Sir2-1 / +) did not result in significantly elevated *dSir2* levels, but both Sir2-3 / + and Sir2-4 / + exhibited significantly increased *dSir2* mRNA levels (~ 2-fold increase over w^1118^*dSir2* levels). This result suggests that the life span extension observed in the control condition of the Tubulin-Gene Switch>Sir2-3 and Sir2-4 life spans may be due to uninduced expression of the *dSir2* construct under some conditions.

In flies from the same cohort as those used for qPCR, we tested whether *dSir2* expression from the UAS-dSir2 transgenes in the absence of a Gal4 driver was sufficient to extend life span (Fig. 1E). A single copy of Sir2-1 that did not show elevated *dSir2* mRNA levels did not extend lifespan, whereas one copy of either Sir2-3 or Sir2-4 did increase lifespan, compared to the genetically matched w^1118^ controls. These results suggest that moderately increased *dSir2* expression, here from un-driven UAS-dSir2 constructs, can extend lifespan in flies.

### dSir2-mediated life span extension is dose-dependent

**Figure 2:**
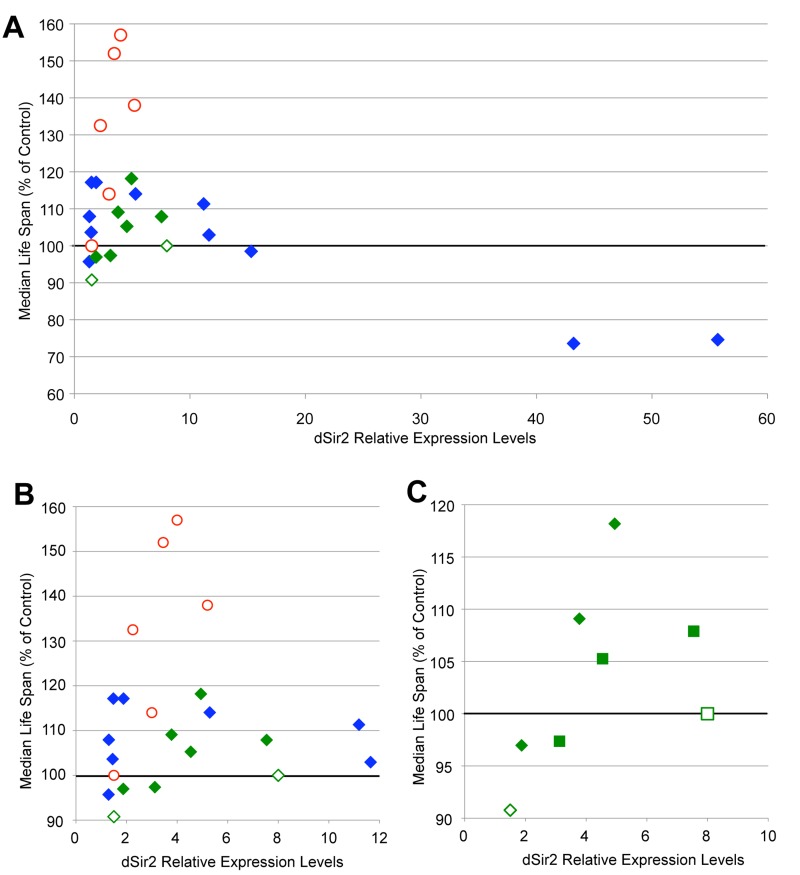
Increased *dSir2* expression extends life span dose-dependently (**A**) Median life span change (shown as % of control) is plotted as a function of *dSir2* expression levels for life span experiments described in this study and in previous reports. (**B**) A finer view of x-axis *dSir2* levels below 12-fold is shown. For all figures, previously published life spans are shown as open data points [5,6,7,10]. For figures **A** and **B**, life spans using dSir2^EP2300^ are shown as red circles and UAS-dSir2 transgene life spans are shown as diamonds. Life spans using the UAS-Sir2-myc lines from [10] are shown in green, while life spans conducted using UAS-Sir2-1, Sir2-3, or Sir2-4 lines in this study are shown in blue. (**C**) *dSir2* expression levels and median life span extension from [10] (diamonds = Myc2, squares = Myc9, open data points indicate data from [10]) and from our studies using the Tubulin-Gene Switch driver and three levels of RU-486 (100 μM, 200 μM, and 500 μM) (green diamond = Tubulin-Gene Switch>Myc2, green square = Tubulin-Gene Switch>Myc9). In life span experiments published in [5], additional EP lines that over-express *dSir2* from the endogenous locus also extended life span, but are not included in this graph because of the lack of data on their level of *dSir2* expression. Life span statistics can be found in table S1, and qPCR p-values in table S2.

The extension of life span seen when *dSir2* expression is moderately increased led to the hypothesis that there may be a dose-dependent element to the effect of *dSir2* expression on life span. Previous studies showing that increased *dSir2* expression extends life span used fly lines that expressed *dSir2* from its endogenous locus at relatively modest levels (~3-5 fold increase) [5,6]. We noted that the flies with increased *dSir2* expression from the Sir2-3 and Sir2-4 transgenes express *dSir2* to a similar extent, from 2-5 fold increased, when life span is extended, while some of the conditions that we had tested had much higher levels of *dSir2*, and rarely extended life span. We noted that the recent study demonstrating no effect of increasing *dSir2* on life span reported that their induced *dSir2* levels were either 1.5- fold or 8-fold increased [10]. In order to examine whether increased *dSir2* expression might have a dose-dependent effect on life span, we plotted *dSir2* expression levels and life span extension from both our studies and those of published life spans (Fig. 2A and B).

As can be seen in Fig. 2, increased *dSir2* expression is associated with life span extension when mRNA levels are elevated approximately 2 to 5-fold compared to controls. In addition, very high *dSir2* expression (greater than 30-fold increase) is associated with shortened life span, suggesting that such high levels of *dSir2* may have a toxic effect. Interestingly, increased expression of *dSir2* in the dSir2^EP2300^ line extends life span more reliably and to a greater extent than induction at similar levels from the UAS-dSir2 transgenes. This suggests that the presence of endogenous regulatory elements may be beneficial. Given that all of the *dSir2* expression levels for flies studied in [10] were outside the predicted optimal range that we report here, we hypothesized that we should be able to extend life span with these transgenic flies if *dSir2* expression within our predicted optimal window could be achieved.

To test this hypothesis we used the Gene Switch system to increase expression of *dSir2* from the myc-tagged UAS-dSir2 transgenic fly lines used in [10]. The Tubulin-Gene Switch driver permitted us to express *dSir2* in the same tissues as in the original study, and to limit the expression to only adult life, avoiding any potential deleterious effects of increased *dSir2* during development. At the same time, this system allowed us to vary the concentration of RU-486 in the food, thereby titrating *dSir2* expression levels in these flies. We used three concentrations of RU-486 (100, 200, and 500 μM) to induce *dSir2* expression, and measured the *dSir2* transcript levels for each of these conditions. We then plotted the median life span increase for both our own new life spans and those previously published with these constructs as a function of the measured *dSir2* levels (Fig. 2C). Life span was extended to the greatest extent when *dSir2* was expressed at moderate levels. These results demonstrate a dose-dependent response for increased *dSir2* expression on fly life span, even for transgenic constructs previously reported not to extend life span.

### High levels of dSir2 expressed from transgenes induce cellular toxicity and dnaJ-H expression

The UAS insertion in the dSir2^EP2300^ over-expression line is located within the 5' UTR of *dSir2*, oriented in a direction so as to specifically drive expression of *dSir2* and not the adjacent gene *dnaJ-H* [9]. Although one study concluded that increased *dSir2* transgene expression is lethal and that *dnaJ-H* suppresses this phenotype [12], in a subsequent study using the same dSir2^EP2300^ line driven by ELAV-Gene Switch at two different doses of RU-486, a dose-dependent increase in life span was observed coincident with increased *dSir2* but not *dnaJ-H* levels [6]. Similarly, a life span extending increase in *dSir2* expression from dSir2^EP2300^ in fat body using the S_1_-106 Gene Switch driver showed no increase in *dnaJ-H* expression [7]. These conflicting studies led us to test whether the expression of *dnaJ-H* in the eye might be unrelated to expression of *dSir2* by the dSir2^EP2300^ EP element.

**Figure 3:**
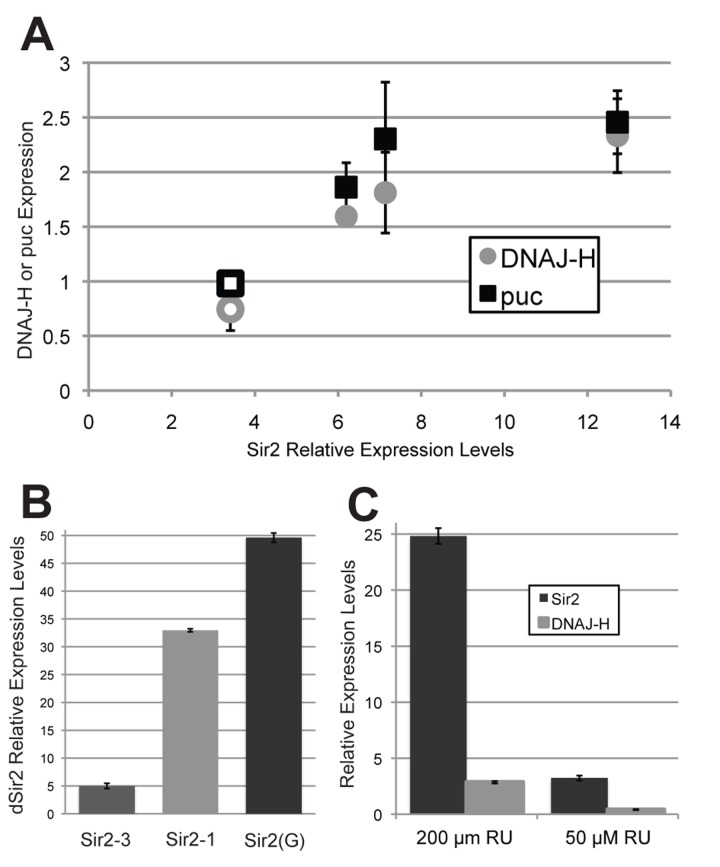
High levels of *dSir2* induce both *puc phosphatase* and *dnaJ-H* expression, but lower levels of *dSir2* expression do not (**A**) Expression of *dSir2* at high levels using the constitutive neuronal driver Elav-Gal4 leads to *dnaJ-H* induction when expression is high, even when expression is not from the native *dSir2* site (filled diamonds = UAS-dSir2 transgenes, open diamond = dSir2^EP2300^). Levels of *dSir2* expression that induce *dnaJ-H* also induce expression of *puc phosphatase*, a JNK-signaling target gene (filled squares = UAS-dSir2 transgenes, open square = dSir2^EP2300^). (**B**) On 500 μM RU-486, the neuronal inducible ELAV-Gene Switch driver and *dSir2*(G) transgene from [12], induces *dSir2* at levels that are extremely high, even when compared to a highly expressing line from our own studies (Sir2-1). (**C**) When the Sir2 (G) transgene is driven by the ubiquitous inducible Tubulin-Gene Switch driver at two different concentrations of RU-486, it can be seen that a high level of *dSir2* expression (with 200 μM RU-486) induces *dnaJ-H* expression, while a lower level of expression (with 50 μM RU-486) does not. Error bars represent SD of 3 biological replicates. p-values can be found in table S3.

We measured *dnaJ-H* and *dSir2* transcript levels in the heads of ELAV-Gene Switch>dSir2^EP2300^ flies and observed that induction of dSir2^EP2300^ from the native *dSir2* locus as in [5] leads to a 3.5-fold increase in *dSir2* and no increase in *dnaJ-H* levels (Fig. S2). We also measured *dSir2* and *dnaJ-H* transcripts in three of our own UAS-dSir2 transgene containing fly lines (Sir2-1, Sir2-3, and Sir2-4), driven by the ELAV-Gal4 driver (Fig. 3A). Expression of *dnaJ-H* is increased in some of the UAS-dSir2 transgenic induced expression conditions but only when the level of *dSir2* induction is greater than 6-fold (Fig. 3A, diamonds).

In order to explore the mechanism by which *dnaJ-H* is elevated in response to transgenically expressed *dSir2*, we measured the level of expression of the JNK signaling gene, puc phosphatase (*puc*), in these conditions and found that *puc* follows the same pattern as *dnaJ-H*, exhibiting increased expression when *dSir2* reaches high levels (Fig. 3A, squares). Thus, expression of *dSir2* at high levels may induce *dnaJ-H* as part of a cellular stress response, not because it is located adjacent to *dSir2*, as was suggested in a previous report [12]. Importantly, moderate levels of increased *dSir2*, such as those typically seen under conditions associated with life span extension, do not induce *dnaJ-H* expression.

These results led us to consider that the lethality previously observed in [12] might have resulted from toxically high levels of *dSir2*. Since crossing the UAS-dSir2 line from that study (referred to here as Sir2(G)) [12] to constitutive GAL4 drivers results in developmental and cellular lethality, we used the ELAV-Gene Switch driver to induce expression from the Sir2(G) transgene in neurons for 10 days during adult life, and measured *dSir2* levels in fly heads. We compared *dSir2* expression from the ELAV-Gene Switch>Sir2(G) flies with ELAV-Gene Switch>Sir2-1 and ELAV-Gene Switch>Sir2-3 flies under the same conditions, and found that Sir2-3, which has moderate levels of increased expression, and Sir2-1, which shows very high *dSir2* levels, both express *dSir2* at much lower levels than the Sir2(G) line, which showed a 50-fold increase in mRNA levels under these conditions (Fig. 3B). We conclude that the lethality of the Sir2(G) transgene resulted from extremely high *dSir2* levels.

Since the Sir2(G) transgene expresses *dSir2* at very high levels, and *dnaJ-H* is elevated in our highly expressing *dSir2* transgenes, we would predict that under conditions of high *dSir2* induction, the Sir2(G) transgene should also induce *dnaJ-H*. Additionally, we expect that induction of *dSir2* expression from Sir2(G) at lower levels should not induce *dnaJ-H*. To test this, we crossed the lethal Sir2(G) line to the ubiquitous Tubulin-Gene Switch driver and induced *dSir2* at different levels by using two different doses of RU-486 (Fig. 3C). At a low concentration of RU-486 (50 μM), *dSir2* is increased 3-fold and *dnaJ-H* levels are not elevated, while at a higher concentration of RU-486 (200 μM), *dSir2* is induced 25-fold over control levels and *dnaJ-H* expression is increased almost 3-fold. Therefore, the Sir2(G) transgene induces *dnaJ-H* induction when *dSir2* levels are high, but not when *dSir2* is slightly induced.

Finally, we tested whether *dnaJ-H* induction was specific to elevated *dSir2* by expressing the foreign GFP mRNA using the strong, ubiquitous, constitutive *daughterless-Gal4* driver and measuring *dnaJ-H* levels (Fig. S3). Interestingly, expressing GFP with this driver led to a 2.5-fold increase in *dnaJ-H*. The induction of a strong *dnaJ-H* expression response when proteins other than *dSir2* are expressed indicates that the induction of *dnaJ-H* when *dSir2* is highly over-expressed is likely part of the cellular stress response itself, and not due to a specific function of *dSir2*.

## DISCUSSION

Here we show that in *Drosophila*, increased expression of *dSir2* extends life span in a dose-dependent manner, thereby resolving apparent controversies in the field about the role of sirtuins in fly aging. By measuring life span while directly titrating the increase in *dSir2* expression through use of a series of new and available UAS-dSir2 transgenes, and by determining the level of *dSir2* expression under conditions for previously published life span studies, we show that when *dSir2* expression is increased to moderate levels (approximately 2-5 fold increased over normal), life span is consistently extended. Expression below this range (less than 2-fold increase), or slightly above it (between 5-10 fold increase) inconsistently extends life span, while higher levels of expression are detrimental to life span and can induce JNK signaling and *dnaJ-H* expression.

Consistent with our findings that the dose of *dSir2* is important in longevity determination, previous work has shown that a reduction in *dSir2* levels shortens life span [13]. Our demonstration that the dose of *dSir2* is critical to life span extension suggests an explanation for the contrary findings reporting an inability to detect life span extension when using dSir2^EP2300^ or two new UAS-dSir2 transgenes [10]. In that report [10], *dSir2* expression from the Sir2-myc2 and dSir2^EP2300^ transgenes were both reported to be below the critical level we determined is necessary for life span extension (less than 1.5-fold increase), while the Sir2-myc9 transgene was expressing at a level of expression that is likely too high (~8-fold) to consistently extend life span. To test the hypothesis that the optimal range for *dSir2* expression was not achieved in this study [10], we used these same two lines (Sir2-myc2 and Sir2-myc9) in conjunction with the conditional Gene Switch system to increase expression of *dSir2* between 2 and 5-fold. In this report, we demonstrate that when *dSir2* expression is increased to within an optimal range, both of these UAS-Sir2 transgenes extend life span compared with their genetically identical sibling cohorts in which *dSir2* is not increased (Fig. 2C).

We note that induction of *dSir2* from its native locus using the dSir2^EP2300^ insertion allele extends life span more reliably and to a greater extent than increased expression from the *dSir2* transgenes at other genomic locations. This may be due to the lower level of *dSir2* induction seen with dSir2^EP2300^, but it could also be due to the presence of favorable endogenous regulatory elements that are maintained when *dSir2* is expressed from its native genomic location.

In a previous study, an increase in *dnaJ-H* was observed when dSir2^EP2300^ was used to increase expression of *dSir2* in the eye using a GMR-Gal4 driver [12], indicating that Gal4 induction of dSir2^EP2300^ may increase expression of both *dnaJ-H* and *dSir2* due to their overlapping genetic locus and that increased co-expression may account for the observed life span extension. However, it has subsequently been shown that *dnaJ-H* is not induced when dSir2^EP2300^ is used to increase expression of *dSir2* in life span-extending conditions in adult neurons [6] or in adult fat body cells [7]. Furthermore, we found that *dnaJ-H* is induced when *dSir2* or GFP is highly expressed from transgenic lines that are not located near the native *dSir2* / *dnaJ-H* genomic locus. The *dSir2* expression conditions that exhibited an increase in *dnaJ-H* levels also showed elevated transcripts of *puc phosphatase*, a target of JNK signaling. Taken together, these results show that increased expression of *dSir2* can induce JNK signaling/puc phosphatase as previously reported, but only when *dSir2* is expressed at high levels.

Our observation that high levels of *dSir2* over expression is toxic fits with previously published results showing that expression of *dSir2* from a transgene induced lethality and activated JNK signaling [12]. In this previous publication, it was reported that any increased expression of *dSir2* from a transgene distant from the native *dSir2* site is lethal [12]. However, the system used in that study consistently induces *dSir2* expression at very high levels, indicating that lethality occurred because *dSir2* was induced too strongly.

The finding that high levels of *dSir2* expression can lead to cytotoxicity is perhaps not surprising given the many known interacting partners of *dSir2*, including proteins central to metabolism (FOXO), mitochondrial biogenesis (PGC-1ɑ), and genomic defense (p53, Ku70) [1]. Additionally, it has been shown that high level expression of the yeast Sir2p from a high-copy plasmid is toxic [14]. It is important to note that lethality in a previous study [12] was shown and measured using the eye-specific GMR-Gal4 driver, which has previously been shown to induce mild developmental defects and apoptosis in the *Drosophila* eye, even without induction of expression from another gene [15]. This suggests that the GMR-Gal4 system that was used to demonstrate lethality may have already been partially sensitized to apoptotic phenotypes.

We conclude that increasing *dSir2* expression in *Drosophila* can extend life span, but caution that experiments testing the overexpression of *dSir2* should ensure that *dSir2* levels are increased to a sufficient extent to induce a positive effect on life span, but not high enough to induce cytotoxicity. The conflicting reports in the literature over whether increased *dSir2* expression extends life span are resolved when the dosage of *dSir2* is taken into consideration.

## METHODS

### *Drosophila* Stocks and Maintenance

All flies were maintained at 25°C in a temperature-controlled incubator at 50% humidity with a 12-hour light/dark cycle. The Tubulin-Gal4 (5138), ELAV-Gal4 (Bloomington 458), and Daughterless-Gal4 (Bloomington 5460) lines were from Bloomington Stock Center. ELAV Gene Switch was a gift from H. Keshishian (Yale University, New Haven, CT). dSir2^EP2300^ from the Bloomington stock center was backcrossed 9 times into the w^1118^ control line before use in qPCR studies. The lethal UAS-dSir2(G) line was a kind gift from KT Min (Indiana University, Bloomington, IN). The Tubulin Gene Switch and Myc-tagged UAS-dSir2 lines (Myc2 and Myc9) were kind gifts from S. Pletcher (University of Michigan, Ann Arbor, MI).

### Construction of UAS-dSir2 lines

To generate the UAS-dSir2 lines (Sir2-1, Sir2-4 and Sir2-4), full length *dSir2* was cloned from an adult *Drosophila* cDNA library into pDONR221 (Invitrogen) and then the pTW vector (Drosophila Gateway Vector Collection, Carnegie Institute) using the Gateway system. Primers used were *dSir2*-GW-F: GGGGACAAGTTTGTACAAAAAAGC AGGC TGCACCATGATGGAAAATTACGAGGAA, and *dSir2*-GW-R: GGGGACCACTTTGTACAAGAA AGCTGGGTCCACTGCTGCTAACTGTCCTGG, amplifying a 2.4 kb fragment. Embryo injection of pTW-*dSir2* into the w^1118^ background was performed by Bestgene, Inc. (Chino Hills, CA). Flies were selected for germline transformation, and kept as homozygous stocks. Different lines were established from three separate integration events.

### Life span Assays

All life span experiments were performed on food containing 150 g/L sucrose, 150 g/L autolysed yeast, 20 g/L cornmeal, and 20 g/L agar, all w/v. Life span experiments using UAS-Sir2 Myc2 or UAS-Sir2 Myc9 lines were performed on food containing 150 g/L dextrose, 150 g/L autolysed yeast, 20 g/L cornmeal, and 20 g/L agar, all w/v. Life span experiments using Gene Switch drivers were conducted on food containing these ingredients plus either RU-486 (Cayman Chemical, Ann Arbor, MI) in ethanol at the stated concentration, or the diluent alone (20 ml/L ethanol) for the control. Flies were collected under light CO_2_ anesthesia, randomly divided into treatment groups, and housed at a density of 25 males and 25 females per vial. Flies were passed onto fresh food every other day (Experiments labeled “EOD” in Table S1) or every day (labeled “ED” in Table S1), and the number and sex of dead flies recorded.

### RNA Isolation and qPCR

Flies for qPCR analysis were collected in a 24-hour window and grown under the same conditions as flies in life span experiments. They were passed onto new food every other day (or every day for life spans passed every day), and on day 10, were snap-frozen in liquid nitrogen and stored at −80°C. For mRNA isolation, 30 whole male flies (for ubiquitious driver experiments) or male fly heads (for ELAV-Gal4 and ELAV-Gene Switch experiments) were used to isolate mRNA using the Dynabeads mRNA DIRECT kit (Life Technologies). mRNA was reverse transcribed using the iScript cDNA Synthesis Kit (Bio-Rad). 50 ng of cDNA was used as qPCR template. qPCR was performed on a ABI 7500 FAST Real-Time PCR machine, using SYBR Green PCR Master Mix (Life Technologies). Each qPCR run was performed using three biological replicates in triplicate. Primer sequences used for amplification can be found in Table S4. GAPDH was used as a normalizing gene.

### Statistical Analysis

All life spans were analyzed in a Log Rank test using the Survival package in R. Student's t-test was used to analyze the statistical significance of qPCR data.

## SUPPLEMENTARY FIGURES AND TABLES


