# Supplementary material for: Increased expression of Drosophila Sir 2 extends life span in a dose-dependent manner
Source: Aging (Albany NY). 2013 Sep 7;5(9):682–91. doi: 10.18632/aging.100599 (PMC3808700; doi:10.18632/aging.100599)
Supplement: Supplementary file 1 [file aging-05-682-s001.pdf]

disease: selectivity, synergy and modulation of protein solubility in Drosophila. Hum Mol Genet. 2000; 9:2811-2820.

12. Griswold AJ, Chang KT, Runko AP, Knight MA, & Min KT. Sir2 mediates apoptosis through JNK-dependent pathways in Drosophila. Proc Natl Acad Sci USA. 2008; 105:8673-8678.

13. Astrom SU, Cline TW, and Rine J. The Drosophila melanogaster sir2(+) Gene Is Nonessential and Has Only Minor Effects on Position-Effect Variegation. Genetics. 2003; 163:931-937.

14. Holmes SG, Rose AB, Steuerle K, Saez E, Sayegh S, Lee YM, Broach JR. Hyperactivation of the silencing proteins, Sir2p and Sir3p, causes chromosome loss. Genetics. 1997; 145:605-14.

15. Kramer JM and Staveley BE. GAL4 causes developmental defects and apoptosis when expressed in the developing eye of Drosophila melanogaster. Genet Mol Res. 2003; 2:43-47.

SUPPLEMENTARY FIGURES

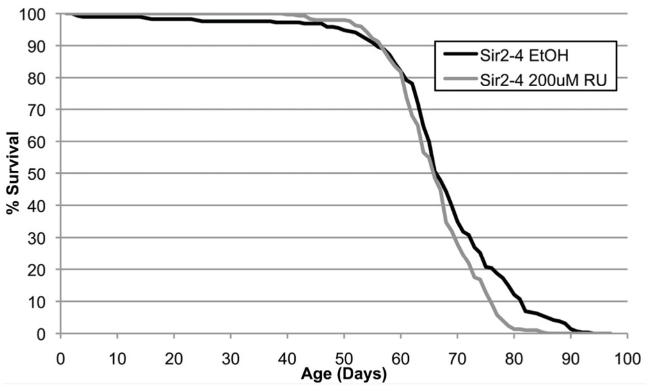

Supplementary Figure 1. Life span is not extended in Tubulin-Gene Switch>Sir2-4 flies in the presence of RU-486. The induction of Sir2 in this condition did not extend life span, indicating that the level of dSir2 expression was not able to extend life span.

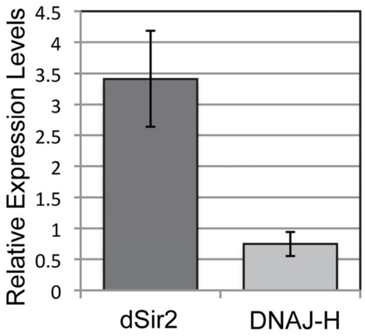

Supplementary Figure 2. *dnaJ-H* levels are not increased in ELAV-Gal4>dSir2^{EP2300} flies over-expressing *dSir2* from the endogenous *dSir2* locus. While dSir2 is over 3-fold elevated in dSir2^{EP2300} flies (dark grey bar represents *dSir2* levels as measured by qPCR, $p<0.0001$), *dnaJ-H* levels do not change (light grey bar represents *dnaJ-H* levels as measured by qPCR, $p=0.34$). Error bars represent SD of 3 biological replicates.

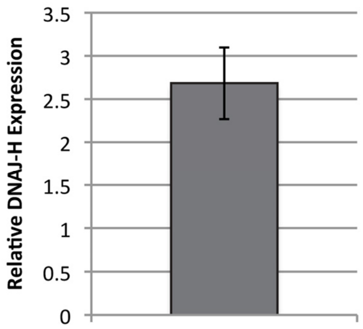

Supplementary Figure 3. When UAS-GFP is expressed using the strong, ubiquitous Daughterless-Gal4 driver, *dnaJ-H* levels are increased ($p=0.0021$). Error bars represent SD of 3 biological replicates.

SUPPLEMENTARY TABLES

Please browse full text version to see the Supplementary tables of this manuscript.
